# Exploring the determinants of health in the aging population: the key role of education and socioeconomic context

**DOI:** 10.3389/fpubh.2026.1659900

**Published:** 2026-02-26

**Authors:** Marco Alberio, Alice Lomonaco, Paolo Pasetti, Susi Anny Veloso Resende, Loris Vergolini

**Affiliations:** 1 Department of Sociology and Business Law, University of Bologna, Bologna, Italy; 2 Department of Political and Social Sciences, University of Bologna, Bologna, Italy; 3 FBK-IRVAPP, Trento, Italy

**Keywords:** economic strain, education, healthy aging, Italy, lifestyles

## Abstract

**Introduction:**

Population aging represents a major demographic transformation in Italy, characterized by higher levels of education and socioeconomic resources compared to previous generations. Education is widely recognized as a key determinant of health, yet the mechanisms linking educational attainment to health in later life remain only partially understood. This study examines the association between education and self-perceived health among older adults in Italy, focusing on the mediating role of economic strain and lifestyle factors, and exploring differences by age, geographical area, and gender.

**Methods:**

We use pooled cross-sectional data (2013–2019) from the ISTAT Aspetti della vita quotidiana survey regarding individuals aged 55 and older. Self-perceived health is analyzed using logistic regression models and mediation analysis is conducted using the KHB method to decompose the total effect of education into direct and indirect effects operating through economic strain and lifestyle variables (alcohol consumption, smoking, and physical activity). Subgroup analyses are performed by age group, geographical area, and gender.

**Results:**

Higher educational attainment is significantly associated with a greater probability of reporting good health. The educational gradient remains robust after controlling for sociodemographic characteristics. Economic strain and physical activity emerge as the main mediators, jointly explaining approximately one-quarter of the total educational effect. Smoking and alcohol consumption play a limited mediating role. The mediating effect of economic strain is stronger among individuals aged 55–64, while the role of physical activity increases with age.

**Discussion:**

Education plays a central role in shaping health inequalities in later life, both directly and through material and behavioral pathways. Policy interventions should address economic vulnerabilities and promote healthy lifestyles, adopting region-sensitive strategies to reduce health disparities in an aging society.

## 1 Introduction

The demographic trend of an increasingly aging population in Italy also implies a paradigm shift, characterized by higher levels of prosperity and educational attainment than those of previous generations. According to the 2023 population and household projections made by ISTAT, 23.8% of the total Italian population was aged 65 or older, representing almost a quarter of the country’s population. Indeed, it is projected that this percentage will exceed one-third of the population by 2050, with Italians aged 65 and older expected to account for 34.5%. These figures underscore the necessity to understand the challenges faced by this age group, particularly those pertaining to health. This approach facilitates a nuanced understanding of the challenges raised by these demographic trends, transcending the broad discourse often associated with the “boomer generation” and its presumed privileges.

In the context of an aging global and local population, scientific evidence highlights the association between higher educational levels and lower mortality, as well as fewer health problems (1–4). Previous studies have demonstrated that educational status exerts a significant influence on health conditions and perceptions of health among the general population, and among the older population in particular [see (5–9)]. This phenomenon has been observed in diverse contexts, including health transitions (9), analyses of subjective perceptions of well-being and health (5, 8), and examinations of objective health conditions (7). Furthermore, an additional body of literature, which focuses on the interplay of macro-, meso-, and micro-level factors, demonstrates that factors such as gender, age group, and region of residence are also crucial when considering health inequalities (4, 10–13).

Despite having one of the highest longevity rates in Europe, Italians generally perceive their health as poorer than older individuals in Northern European countries. In Southern European countries, self-perceived health is lower than in Scandinavian and Anglo-Saxon countries, and patterns of health transitions also differ (9, 38). In Italy, research indicates that patterns of health inequality are aligned with socioeconomic status, particularly educational attainment (3, 39). Despite the presence of a contemporary healthcare system in Italy, health perceptions among the older adults continue to be significantly influenced by socioeconomic disparities within Italian society (3). This finding indicates that, despite their longevity, Italians’ experience and perception of their good health may be shaped by factors such as lifestyle, thereby rendering the aging process multifaceted and unevenly experienced among individuals. (5). In the Italian context, although the literature has already examined how education influences health status (14, 15), few studies have focused specifically on the older adults and how intervening variables (e.g., lifestyle and socioeconomic resources) influence self-perceived health among this group.

This paper analyzes the relationship between education and health among older Italians by means of data from ISTAT’s *Aspetti della vita quotidiana* survey (2013–2019). The following three research questions have been identified: (i) the influence of educational level on health perception among the population under study; (ii) the mediating effect of intervening variables related to education, such as lifestyles and economic resources, on health perception; (iii) the change in mediation effects according to age, geographical area of residence and gender. It is therefore vital to understand the dynamics behind the current health conditions of the older population to assess present levels of well-being and to inform future public policies for a continually aging population.

## 2 Health and sociodemographic factors

### 2.1 Education

A substantial body of research shows that higher educational levels correlate with better self-perceived health, lower comorbidity risks, and reduced mortality rates [e.g., (1, 2–6, 15)]. This relationship is typically explained by two factors: how education affects lifelong habits (diet, alcohol consumption, smoking, physical activity) and how it provides better structural and material conditions (6).

It is widely documented that older people who have attained higher levels of education generally enjoy a more favorable economic situation. This, in turn, enables them to reside in healthier living environments and to access superior quality medical care and a more balanced diet (5). Furthermore, personal behaviors can be influenced by education, insofar as greater schooling can engender a more profound understanding of the factors that influence their health, which in turn leads them to adopt preventive practices. Several studies have shown that individuals with higher educational attainment generally exhibit healthier behaviors and lifestyles, along with better access to health services, compared to those with lower educational qualifications (16, 17). In accordance with the cumulative advantage hypothesis, which posits that individuals accrue benefits over the course of their lives that positively impact their health, Alperin and Kyzyma (18) posit that individuals with higher levels of education demonstrate superior management of their health and engage in physical exercise with greater frequency in comparison to those with lower levels of education. Furthermore, individuals who were raised in families in which at least one parent had attained a high level of education demonstrated better health throughout their lives (18). This finding suggests that the health of the older adults is also significantly influenced by their earlier life stages, with education emerging as a prominent factor. This observation underscores the notion that inequalities experienced throughout the life course tend to accumulate in later years.

Recent studies conducted within the Italian context are consistent with international research on the links between education and health outcomes, including self-perceived health. Sarti and Spinola (8) highlight that socioeconomic factors, particularly education and occupational status, significantly influence health in both Italy and Argentina. Although their study does not focus exclusively on older adults, they find that education has a stronger impact on mental health among individuals from lower socioeconomic backgrounds in Italy compared to those from the working and middle classes. Similarly, Adjei et al. (40), using data from Germany, Spain, Italy, the United States, and the United Kingdom, confirm that educational attainment is consistently associated with better self-perceived health for both men and women across all countries considered, including Italy.

In a multilevel analysis of older Italians, Pirani and Salvini (3) identify several key determinants of self-perceived health: (1) education and income emerge as the primary drivers, with higher socioeconomic status associated with fewer chronic conditions, healthier behaviors, and more favorable health assessments; (2) women report worse health than men, although this gender gap diminishes after controlling for other variables, indicating that gender differences in health perception are partly compositional; (3) physical activity is positively associated with self-rated health, likely reflecting both its health benefits and the tendency of healthier individuals to remain active; (4) strong social ties contribute positively to perceived health; and (5) although regional differences are modest, more positive perceptions of local healthcare systems are associated with better self-assessed health.

Further supporting this perspective, Maniscalco et al. (7) examine the relationship between self-perceived health and quality of life among the older population in Spain, Greece, and Italy. Their findings indicate that while income is more closely tied to overall quality of life, education is a more critical determinant of self-perceived health, underscoring the distinct nature of these two constructs.

Taken together, these studies reinforce the idea that education plays a key role in shaping subjective health perceptions. However, both self-perceived and objective health outcomes are influenced by a broader array of factors. These include material and structural conditions, social capital, access to healthcare, lifestyle behaviors, social relationships, health literacy, economic inequality, and psychological well-being (8, 19). Accordingly, the determinants of health perceptions can be categorized into sociodemographic, behavioral, and psychological domains.

### 2.2 Gender

The literature on gender differences in health consistently reveals a well-documented paradox: men tend to have shorter life expectancies but report better health, whereas women live longer yet often experience poorer health conditions (5, 9, 10, 12, 20). In this context, gender is not merely a biological category but a social construct that shapes identities, roles, lifestyle habits, disease incidence, healthcare practices, and socioeconomic positioning (11).

Several authors (6, 12) have similarly demonstrated the effect of sex on health outcomes, highlighting that gender differences arise from a combination of biological factors and socially patterned behaviors, with women facing higher risks of comorbidities and disabilities partly due to higher obesity rates and lower levels of physical activity. Crimmins et al. (10) also challenge purely biological explanations for gender disparities in health, underscoring the role of contextual factors such as healthcare infrastructure, disease patterns, and public health systems. While men are more susceptible to severe diseases like heart conditions and diabetes, women more frequently report chronic but non-lethal conditions, including arthritis and depression. These conditions contribute to women’s lower self-perceived health despite their longer life expectancy (21). More recently, Osobsama et al. (20) show that women are more likely than men to use primary healthcare services, whereas men exhibit higher rates of hospitalization.

Focusing specifically on self-perceived health, the main outcome of interest in this study, Ross et al. (13) explore the joint influence of gender and education. Their findings indicate that although both factors independently shape health self-perception, the effect of education is stronger for women. Women with lower levels of education report significantly poorer health perceptions than their male counterparts. However, each additional year of education improves perceived health, and this educational gradient is steeper for women, such that the gender gap narrows considerably among those who have completed high school (usually 18–19 years old).

Crimmins et al. (22), in a comparative study including Italy, finds that women report poorer self-perceived health than men when controlling only for age. However, once the presence of illnesses is considered, the gender gap in health perception becomes statistically insignificant. This suggests that women’s more negative health reports may reflect not only their greater exposure to illness but also the types of conditions they experience, often those leading to disability rather than mortality.

Adjei et al. (40) also document persistent gender differences in health perception among older adults across five countries: Germany, the UK, Spain, Italy, and the United States. Their study shows that Italy displays the widest gender gap in perceived health among the older adults, with differences reaching up to 47%. The authors attribute these disparities to unequal participation in the paid workforce, differences in educational attainment, and varying distributions of leisure and domestic responsibilities between men and women.

These findings are consistent with Pirani and Salvini (3), who underscore the significant role of gender in shaping self-perceived health among Italy’s older population, with women consistently evaluating their health more negatively than men. At the broader European level, Dimitrova (23) also finds that women report worse health than men across nearly all countries included in her study, reinforcing the robustness of this gendered pattern.

### 2.3 Geographical area of residence

Geographical differences represent a critical dimension in understanding health inequalities among the older population. A growing body of research highlights how economic, environmental, and cultural disparities across regions significantly influence population health (41, 42, 43). Curtis (44) and Fan et al. (41) underscore the role of unequal resource distribution in explaining regional health disparities, particularly with respect to access to healthcare, education, and income.

In the Asian context, several studies document marked spatial inequalities in older adults health. Fan et al. (41) identify substantial interprovincial disparities in China, with 2.4% of the variation in older adults health attributable to differences in economic, educational, and medical resources. These disparities are particularly pronounced in less developed rural areas, where individual income gaps further exacerbated inequality. Similarly, Cui et al. (42) observe both inter- and intra-regional health disparities among older Chinese adults, pointing to localized health and social conditions as key contributing factors. Crimmins et al. (10) argue that variations in health and mortality should not be ascribed solely to biological or hormonal differences; instead, they stress the importance of healthcare infrastructure, contextual risk exposures, and the influence of historical and systemic factors.

In Europe, research likewise confirms that geographic and social contexts shape health disparities among older adults. Oksuzyan et al. (21) identify multiple intersecting factors, such as region of residence, health behaviors, literacy, class, and education, that shape gendered health outcomes across countries. In Spain, Fernandez-Martinez et al. (43) report that older adults in the southern regions tend to rate their health more negatively than those in wealthier northern areas, linking these differences to socioeconomic and psychological factors. Poorer perceived health was associated with advanced age, chronic conditions, depressive symptoms, and reduced independence in daily activities. In Greece, Dermartis et al. (38) identify a clear gradient in perceived quality of life by settlement type: older residents in urban areas report the most positive health perceptions, followed by those in semi-urban and rural areas.

In the Italian context, although extensive research has examined regional disparities in healthcare organization and governance (24–27, 39), few studies have directly addressed regional variation in self-perceived health among the older population. Nonetheless, the influence of regional socioeconomic inequalities on health is well documented. Franzini and Giannoni (28) identify regional income disparities as a major driver of health outcomes, advancing two explanatory frameworks: material deprivation, whereby limited economic resources directly impair health, and social-psychological well-being, which emphasizes the role of social capital and the negative impact of social isolation. These dynamics are particularly salient in economically disadvantaged southern regions, where poverty, unemployment, and income inequality converge with reduced access to healthcare to worsen health outcomes. Mortality statistics further reflect these territorial disparities; Lenzi et al. (29) show that treatable mortality varies by gender and region, with men exhibiting higher rates overall, and southern Italy, except for Puglia, recording the highest levels of avoidable mortality.

### 2.4 Research questions

Building on the extensive literature documenting a positive association between education and health in later life, the aim of this study is to clarify both the strength of this relationship and the mechanisms through which it operates among older adults in Italy. Our first objective is to estimate the association between educational attainment and self-perceived health for older individuals in Italy. Accordingly, our first research question is:

*R1*: Is educational attainment associated with self-perceived health among older adults in Italy and how strong is this relationship? Based on previous studies, we expect to find that higher levels of education are associated with a higher probability of reporting good self-perceived health.

Beyond this primary association, our main contribution lies in attempting to open the “black box” of the relationship between education and self-perceived health by considering the role of intervening mechanisms related to health-related behaviors and material conditions. Education may indirectly affect health by reducing economic strain and by promoting the adoption of healthier lifestyles, such as engaging in physical activity and avoiding smoking and excessive drinking (30, 37). This leads to our second research question:

*R2*: To what extent is the association between education and self-perceived health mediated by economic strain and lifestyle factors? We expect to find a mediating effect for these variables; however, we do not have precise hypotheses regarding which mediator is the strongest.

In the previous subsections, the role played by gender and territorial inequalities was highlighted, and it is therefore relevant to explore whether these mediating mechanisms operate differently across population subgroups. Geographical area of residence and gender may shape both access to resources and the adoption of a healthy lifestyle. In addition, differences across age groups cannot be excluded, as our sample consists of individuals aged 55 and older, and the size of this group may conceal significant heterogeneous effects. This motivates our third research question:

*R3*: Do the mediating effects of economic strain and lifestyle vary by age group, geographical area of residence, and gender? We expect to find a significant heterogeneity in economic strain and lifestyles across age groups, geographical areas, and gender. However, we do not formulate precise hypotheses about which mediator or subgroup will exhibit the strongest effects.

## 3 Materials and methods

The analyses presented in this paper are based on a pooled sample of multiple waves (2013, 2014, 2015, 2016, 2017, 2018 and 2019) of the ISTAT *Aspetti della vita quotidiana* (AVQ) survey, an annual survey representative at the regional level. This survey explores several dimensions of social life within Italian households and encompasses the entire Italian population across all age groups. For this study, the final dataset was created by harmonizing and combining the cross-sectional waves, focusing exclusively on individuals aged 55 and older. The analytical sample comprises 94,916 observations and is based exclusively on cases with non-missing information for all variables.

The choice of the AVQ survey is justified because it contains all the information needed to answer our research questions. The dependent variable is health self-perception measured through the question: “How is your health in general?” The response options are: “Very good”; “Good”; “Neither good nor bad”, “Bad” and “Very bad.” In our analysis, this variable has been treated as a dummy variable, taking the value “1” for “Very good” or “Good” and “0” otherwise.

The main independent variable is the educational level of the interviewees, coded in five categories: “No educational qualification”, “Primary”, “Lower secondary”, “Upper secondary” and “Tertiary.” The mediators are economic strain and lifestyle factors. Economic strain is measured through a dummy variable concerning how the household’s overall economic resources were in the last 12 months. This variable takes value “1” if the economic resources were excellent or adequate and “0” otherwise (i.e., scarce or completely insufficient). Regarding lifestyle, we consider: alcohol consumption, smoking habits and physical activity. Alcohol consumption counts the number of glasses of alcohol usually consumed outside meals in a week. Smoking habits is a dummy variable that takes value “1” if the interviewee smokes or has smoked in the past, and “0” if they have never smoked. Physical activity, performed recreationally, is also coded as a dummy variable, taking the value “1” if this activity is performed once or more than once a month and “0” otherwise (i.e., no activity or less than once a month). The descriptive statistics of the variables used in the analysis are reported in Appendix (Table A1) in Supplementary material.

The analytical strategy is based on two steps and is designed to answer the research questions presented in the previous sections. The first step is intended to quantify the role played by educational level in influencing the self-perceived health status of older adults people in Italy. Since our dependent variable is a dummy variable, we rely on a logistic regression model:


$$\log(\frac{p}{1-p})=\alpha +\text{βEduc}+\mathrm{\gamma X}$$ (1)

where *p* is the probability of reporting good self-perceived health, *Educ* represents the educational level and *X* denotes a set of covariates (sex, age, region of residence, marital status, survey, occupational status). Equation 1 can be enriched by including the mediators (*M*):


$$\log(\frac{p}{1-p})=\alpha +\text{βEduc}+\mathrm{\theta M}+\mathrm{\gamma X}$$ (2)

**Figure 1:**
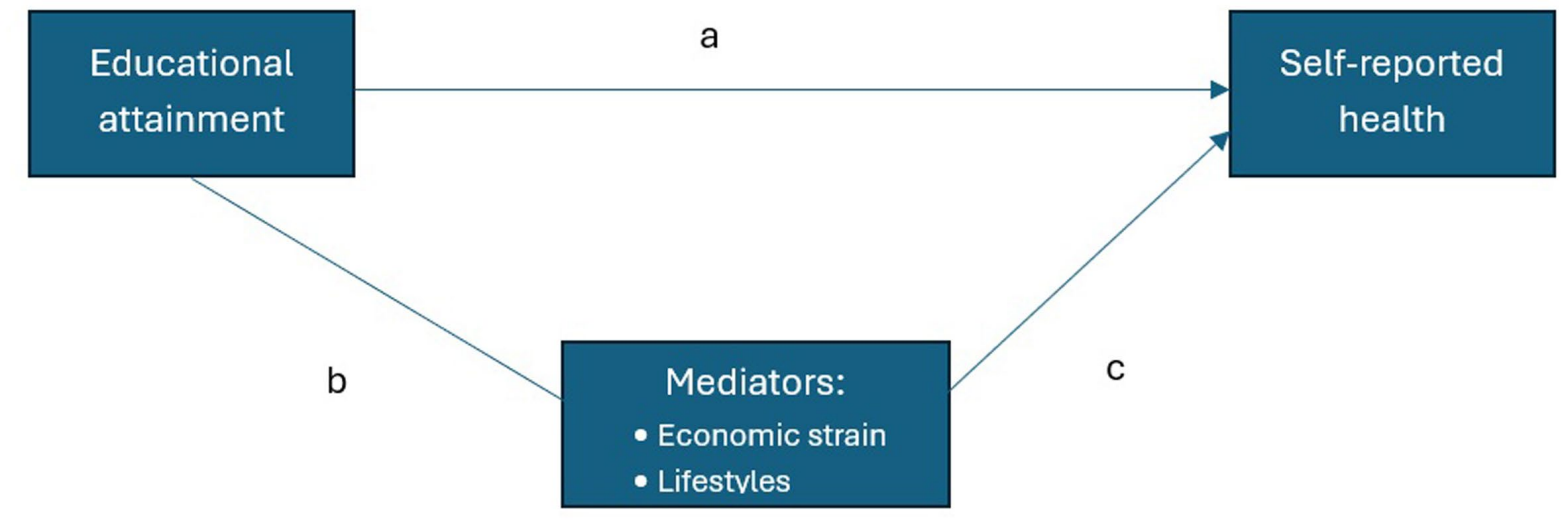
Graphical representation of the relationship between the variables of interest. Path (a) represents the direct effect of educational attainment on self-reported health, whereas paths (b) and (c) capture the indirect effect operating through the mediators (economic strain and lifestyles).

Equation 2 represents the second step of our analyses, and it is used to assess the role of the mediators in influencing both self-perceived health status and the effect of education on this outcome. To enable the comparison between the different models, we present average marginal effects rather than odds ratios or logit parameters (31, 32). In this way, Equation 2 serves to introduce mediation analysis, whose logic is summarized in Figure 1.

Mediation analysis allows the total effect of education to be decomposed into direct and indirect effects, with the latter operating through four potential mediators: economic strain and three lifestyle-related variables (alcohol consumption, smoking habits and physical activity). As noted above, because coefficients from logistic regressions are not directly comparable across models, we apply the KHB method to achieve this decomposition (33).

## 4 Results

The results are presented in two subsections. The first provides descriptive findings on variations in perceived health and health status, including notable regional differences. The second subsection details a series of logistic regression models. These models examine how education influences health self-perception among older Italians, assess the mediating roles of lifestyle and economic conditions, and explore how these relationships vary by geographical area and gender.

### 4.1 Descriptive evidence

**Figure 2:**
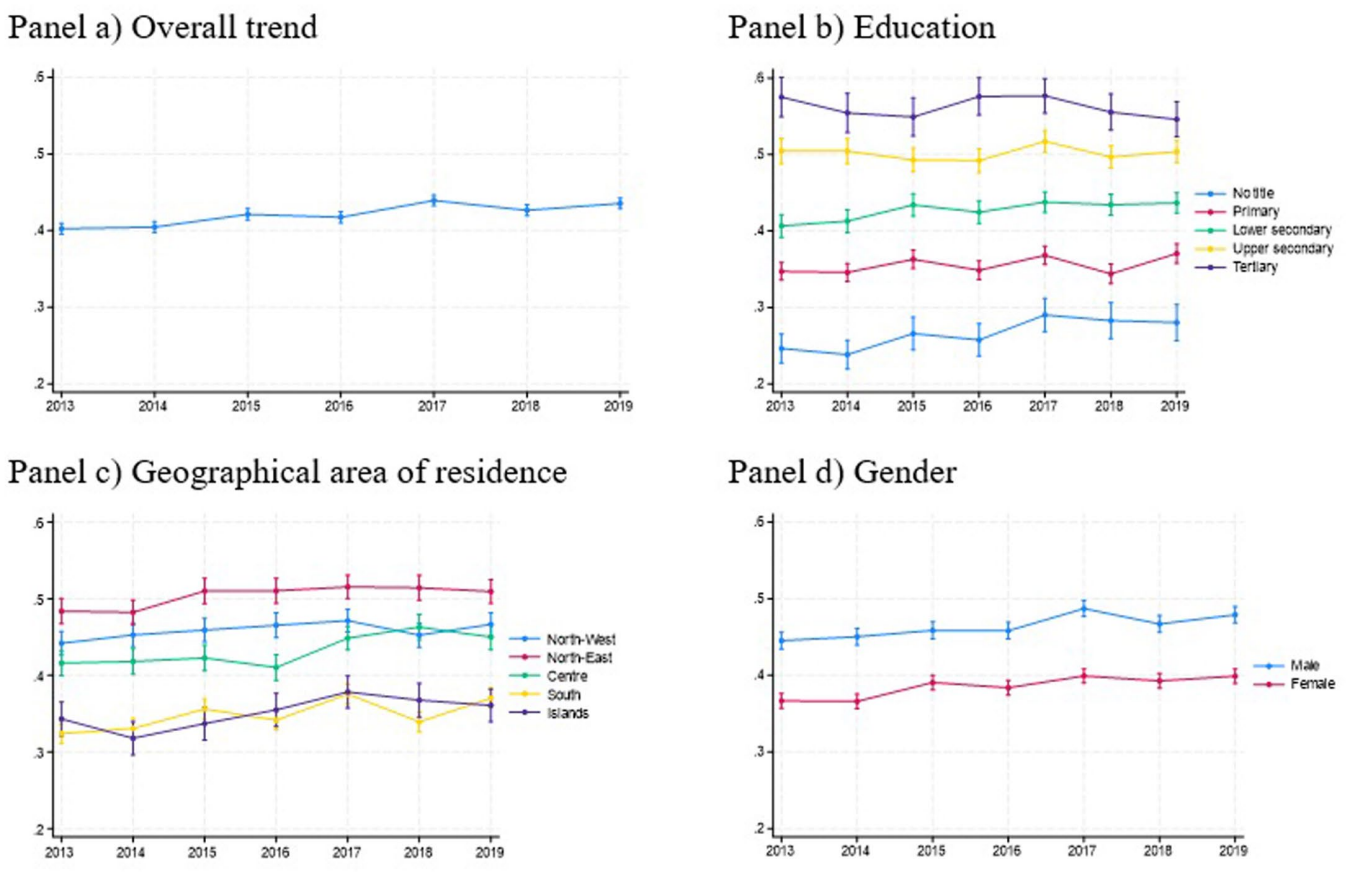
Trend of self-reported health, Italy, 2013–2021. Trend of self-reported health, Italy, 2013–2019. The analyses are controlled by age. **(a)** Overall trend, **(b)** education, **(c)** geographical area of residence, and **(d)** gender.

Figure 2 illustrates trends in self-reported health status from 2013 to 2019, stratified by different characteristics. The general trend (Panel A) shows a gradual improvement in perceived health. While there are slight fluctuations, the overall trajectory suggests a consistent rise in the proportion of individuals reporting good health. More precisely, the share of healthy older people increases from 40% in 2013 to almost 48% in 2021. Panel B highlights stark differences in health self-perception by on educational level. Individuals with tertiary education consistently report the highest levels of good health, followed by those with upper secondary and lower secondary education. Conversely, those with no educational qualifications exhibit the lowest levels of self-reported health. The gap between the lowest and highest educational groups remains significant but stable over time, underscoring the persistent impact of educational inequality on health outcomes.

Regional disparities are evident in Panel C. The North-West consistently reports the highest levels of good health, followed closely by the North-East and the Centre. In contrast, the South and Islands report lower health perceptions, with the Islands (Sicily and Sardinia) consistently showing the lowest levels. These regional differences may be linked to variations in healthcare access, economic opportunities, and socioeconomic determinants of health. While all regions show some improvement over time, the gap between the North and South persists, reflecting ongoing regional disparities.

Panel D, finally, reveals gender-based differences in perceived health. Men consistently report higher levels of good health compared to women throughout the observed period. Although both genders show gradual improvements over time, the gender gap remains stable, suggesting that factors such as differential exposure to health risks, societal roles, and access to healthcare shape these outcomes.

### 4.2 The role of education in self-reported health

After presenting the descriptive data from our analysis, we proceed with a logistic regression analysis to examine how education level influences the self-reported health of the older population. Table 1 reports three models predicting self-perceived health. The first model controls sociodemographic characteristics, the survey year and employment status. Model 2 adds economic status, while Model 3 further includes three lifestyle variables (alcohol, smoking and physical activity). The primary aim is to compare how the coefficients vary relating to the educational qualification across the three models, to assess whether these mediators exert an intervening effect. Methodologically, the estimates presented in Table 1 are derived from logistic regression models and to facilitate the interpretation of the results, the parameters are presented in the form of average marginal effects. In this way, they can be interpreted as differences in terms of percentage points.

**Table 1:** Logistic regression: average marginal effects (AME) for probability of being healthy (selected parameters).

	Model (1)		Model (2)		Model (3)	
	AME	S.E.	AME	S.E.	AME.	S.E.
*Education*						
No title	Ref.					
Primary	0.075^***^	(0.006)	0.069^***^	(0.006)	0.070^***^	(0.006)
Lower secondary	0.121^***^	(0.007)	0.109^***^	(0.007)	0.112^***^	(0.007)
Upper secondary	0.168^***^	(0.007)	0.146^***^	(0.007)	0.154^***^	(0.007)
Tertiary	0.211^***^	(0.009)	0.176^***^	(0.009)	0.191^***^	(0.009)
*Econ. strain*						
Excellent or adequate			Ref.			
Scarce or completely insufficient			−0.095^***^	(0.003)		
*Alcohol (number of glasses)*					0.038^***^	(0.003)
*Alcohol (number of glasses)^2^*					−0.005^***^	(0.001)
*Smoke*						
No					Ref.	
Yes					−0.029^***^	(0.003)
*Physical activity*						
No					Ref.	
Yes					0.093^***^	(0.003)
N	94,916		94,916		94,916	
Pseudo *R*^2^	0.078		0.085		0.087	

*p < 0.10, **p < 0.05, ***p < 0.01. Ref., reference category.

The models control for sex, age, region of residence, marital status, year of the survey, occupational condition. Full models are reported in the Appendix (Table A2) in Supplementary material.

Educational attainment is positively associated with the probability of reporting good health across all models. In Model (1), individuals with primary education are 7.5 percentage points more likely to report good health compared to those with no formal education. This likelihood increases progressively with higher educational levels, reaching 21.1 percentage points for those with tertiary education. While the magnitude of these effects decreases slightly in Models (2) and (3) due to the inclusion of the mediators, the positive and significant relationship remains robust. This underscores the critical role of education in shaping health perceptions, as highlighted by the previous literature.

In Model (2), we introduce the first mediator: economic strain. It is negatively associated with self-perceived good health, as indicated by a significant coefficient of −0.095. This suggests that individuals experiencing economic difficulties are less likely to report being in good health, highlighting the adverse impact of financial stress on well-being.

Regarding lifestyle factors, we rely on three indicators: alcohol consumption, smoking habits and physical activity. Model (3) presents the results for these variables. The relationship between alcohol consumption and self-reported health perceptions is nonlinear. The coefficient for alcohol consumption is positive (0.038), while the squared term is negative (−0.005), indicating that moderate alcohol consumption may be associated with better health perceptions, but excessive consumption has a detrimental effect. Smoking is significantly negatively associated with the probability of reporting good health; the coefficient of −0.030 reflects the well-established health risks associated with smoking, reinforcing its negative impact on self-perceived well-being. Physical activity emerges as the strongest behavioral predictor of self-perceived good health (0.093), highlighting the significant health benefits of regular physical activity, which likely improves physical fitness, reduces stress, and enhances overall well-being.

### 4.3 The role of economic strain and lifestyle factors in self-reported health

The next step of the analysis is to estimate the mediation effects of economic strain and lifestyle factors. Tables 2, 3 quantify this mediation. In Table 2 we estimate four different models, each including a different mediator. For the overall population, the most influential mediators are: economic strain (15.63%) and recreational physical activity (11.49%), whereas smoking and alcohol consumption play a negligible role. The prominent role of economic strain aligns with the argument that material resources are pivotal in shaping health outcomes. Higher education often translates into better socioeconomic positions, providing individuals with greater financial stability and access to (private) healthcare and other resources. Reduced economic strain likely alleviates chronic stress and enables healthier lifestyle choices, indirectly improving health perceptions.

**Table 2:** Mediation effects, percentage of the total effect of the education attainment due to each mediator.

Mediators	% Mediation			
	Overall	55–64	65–74	+75
Economic strain	15.63	22.48	15.58	12.50
Alcohol consumption	0.66	1.09	0.43	0.56
Smoke	2.22	1.45	1.52	1.60
Physical activity	11.49	7.89	11.51	16.19
N	94,916	31,405	29,732	33,779

Each Row represents a different model.

**Table 3:** Mediation effects, percentage of total effect of education due to economic resources and physical activity.

Mediators	% Comprehensive mediation	% Attributable mediation all single variations
Economic strain	27.35	58.12
Physical activity		41.88
*N*		94,916

Among lifestyle factors, physical activity is the most significant mediator, explaining 12% of the total effect. Smoking (2.22%) and alcohol consumption (0.66%) play more minor roles. These findings suggest that education fosters healthier behaviors through increased awareness of health risks and benefits. Educated individuals are more likely to adopt regular physical activity, contributing to better health outcomes. Surprisingly, the mediating roles of smoking and alcohol consumption are very low. This result can be explained by the so-called survivor effect: among older people, those who engaged in heavy smoking or excessive alcohol consumption may already have experienced significant health consequences (i.e., died), potentially leading to selection bias. This means that those remaining in the sample are either moderate users or have successfully changed these behaviors, reducing their explanatory power as mediators. Older individuals may also reduce or cease risky behaviors such as smoking and excessive drinking, either due to age-related health concerns or medical advice.

Table 2 also presents the results of the mediation analysis broken down by age group. The overall picture is confirmed, but some variations emerge across age groups. More specifically, the role of economic strain is greater for the youngest age group (i.e., those aged between 55 and 64). This could be due to the fact that the oldest age group is better protected by pensions, which mitigate economic difficulties compared to younger cohorts. The mediation effect of alcohol consumption is minimal for all age groups, suggesting that its role as a mediator in the relationship between education and health is limited in this context. For smoking, the middle group (65–74) belongs to a cohort with historically higher smoking rates and is likely to have quit more recently. In contrast, the oldest group may have stopped smoking much earlier, or this may reflect some selection, as those who smoked heavily in the past may not have survived to the oldest age group. Regarding physical activity, the mediation effect increases with age. This is plausible for two reasons: firstly, cumulative effects over a lifetime likely play a greater role at older ages; secondly, physical activity helps to maintain or improve mobility, which is particularly important at ages when physical functioning is more vulnerable.

Table 3 considers a model including the two main mediators simultaneously and shows that approximately 27% of the total effect of education is explained by these two variables. The second column of Table 3 reports the weights of the individual mediators, which are consistent with what we saw in Table 2. Overall, our results underscore the crucial role of education in shaping self-reported health through access to resources, and healthier behaviors among more educated individuals. In fact, it is noteworthy to emphasize that a substantial part of the total effect (about 75%) is not explained by the mediators. The remaining 75% includes the direct effect of education as well as any other indirect mechanisms not accounted for in the current mediation models. The direct effect might reflect factors related to cognitive abilities and to stronger social networks. Cognitive abilities may encompass the ability to interpret symptoms, more effectively manage health, and access health information and medical resources (i.e., health literacy). Stronger social networks may provide emotional and practical support, further enhancing health outcomes.

The final step examines how the roles of the mediators can change according to geographical area of residence and gender. Table 4 shows the results broken down by North and Center-South, showing substantial differences in mediation percentages between regions. On average, the role of mediators is stronger in the North. Economic resources are particularly influential in the North, whereas physical activity plays a more prominent role in the South.

**Table 4:** Mediation effects, percentage of total effect of education due to economic resources and physical activity according to geographic area of residence.

Mediators	North		Center-South	
	% General mediation	% Attributable to each mediator	% General mediation	% Attributable to each mediator
Economic strain	35.19	50.56	24.04	36.34
Physical activity		49.44		63.66
*N*	57,111		37,805	

The greater mediation effect observed in the North may reflect better structural conditions that enhance the impact of economic strain reduction. More precisely, educational attainment may more effectively reduce economic strain, while in the South, structural barriers might limit its potential to improve financial stability and health outcomes. Physical activity appears to play a larger mediating role in the South, potentially due to cultural or lifestyle differences. In regions where economic opportunities are limited, educated individuals may rely more on personal health behaviors, such as physical activity, to maintain their health. In other words, the lack of structural support in the South could mean that health improvements rely more heavily on individual actions, rather than systemic factors such as reductions in economic strain.

Table 5 shows the mediation effects by gender. No significant differences were observed, although the mediation effect is slightly larger for males (29.5% vs. 25.3%). The absence of gender differences, at least in the mediation analysis, suggests that, for this older population, the pathways linking education, economic strain, and physical activity to health operate similarly for men and women.

**Table 5:** Mediation effects, percentage of total effect of education due to economic resources and physical activity according to gender.

Mediators	Male		Female	
	% General mediation	% Attributable to each mediator	% General mediation	% Attributable to each mediator
Economic strain	29.54	43.00	25.34	39.77
Physical activity		57.00		60.23
*N*	41,197		53,719	

## 5 Discussion

Considering that the main objective of this study was to examine the relationship between educational attainment and self-perceived health among older adults in Italy (taking into account the mediating role of lifestyle factors and economic resources, as well as variations in these effects by age, region of residence, and gender) we focused our investigation on the following hypotheses: (a) Older adults with higher levels of education report better self-perceived health compared to those with lower educational attainment. (b) Healthier lifestyles and greater economic resources positively mediate the relationship between educational attainment and self-perceived health. (c) The mediating effects of lifestyle factors and economic resources on the relationship between education and self-perceived health vary according to age, geographic area of residence, and gender.

The analysis confirms a strong correlation between educational attainment and self-perceived health among older adults, aligning with previous research (3, 40). Higher levels of education significantly increase the likelihood of reporting good health. Mediation analysis reveals that this relationship is partially explained by two key factors: improved economic conditions and healthier behaviors, such as physical activity and avoiding harmful habits, underscoring the multifaceted role of education in shaping health outcomes later in life.

However, approximately 75% of the total effect of education on self-perceived health remains unexplained by the mediators considered in this study. While not empirically tested here, literature suggests several plausible additional mediators such as social capital, health literacy, and psychological well-being, often associated with greater economic security (8).

A subsequent analysis of mediation effects by age group revealed notable differences. Specifically, younger individuals appear to be more affected by economic strain than older cohorts. One possible explanation is that older adults may benefit from the relative financial security provided by pension systems, particularly those based on the more generous retributive model in place in the past (34, 35). This system, which calculated pension benefits based on previous earnings, may have shielded older cohorts from current economic hardships to a greater extent than younger individuals, who face less favorable pension conditions and more precarious labor market trajectories (21, 34, 35). With regard to healthy habits, particularly physical activity, its mediating effect becomes more pronounced with advancing age. This finding is consistent with the cumulative effect of physical activity across the life course, impacting self-perceived health more significantly as mobility preservation becomes crucial in later life (18).

The final component of our analysis explored how the effects of the identified mediators vary by geographic area of residence and gender. Regarding regional disparities, our findings both corroborate and extend the existing literature. While previous studies on the general population have highlighted territorial inequalities in health conditions and health perceptions (23, 43, 45), our results reveal important nuances specific to the older population. We found that economic resources play a more prominent mediating role in the northern regions, whereas physical activity emerges as a more significant factor in the southern regions. This pattern likely reflects the documented economic vulnerability and more restricted access to healthcare services in southern Italy. In such contexts, physical activity may take on a compensatory role, partially offsetting the adverse effects of limited material and institutional resources and thereby exerting a stronger influence on self-perceived health.

Regarding gender differences, this study indicates no significant variation between men and women in the effects of the analyzed mediators. While overall gender disparities in health perception are well documented (13, 22, 40) and confirmed in our analyses (see Table A2 in Supplementary material), these differences dissipate when focusing on mediating factors. This suggests that economic strain and lifestyle, particularly physical activity, exert comparable influences on health perception across genders, thus not accounting for the aggregate gender gap. These novel findings point to the potential relevance of alternative mediators, such as psychological well-being, social capital, and other education-related resources, in shaping gendered self-perceived health patterns.

## 6 Final remarks and conclusions

While these findings generally align with the existing literature, our analysis uncovers several important nuances specific to the Italian context. A key observation is that self-perceived health declines with age, with individuals in the oldest age groups reporting significantly poorer health. This underscores the critical importance of maintaining and strengthening healthcare services throughout the life course, and particularly during older age. Additionally, marked geographic disparities emerge. Older adults residing in the South and the Islands report significantly more negative health perceptions than their counterparts in the Central and Northern regions. However, it is also noteworthy that, over time, health self-perceptions among Italians have generally improved over time.

With regard to education, our findings indicate that its interaction with age does not mitigate the overall trend of declining health perception in older age. Nevertheless, individuals with only primary education or no formal qualifications consistently report worse health perceptions, highlighting the persistent disadvantage associated with low educational attainment. In contrast, no significant differences are observed among individuals with secondary or higher educational qualifications. Our analyses confirm that education influences health self-perceptions primarily through indirect pathways, specifically through improved economic conditions and healthier lifestyles, including physical activity. Our study’s novel contribution lies in demonstrating that the salience of these mediating mechanisms varies geographically across Italy.

The results of this study carry both theoretical and practical implications. Theoretically, our findings reinforce the existing literature by demonstrating that education, as a fundamental resource, helps mitigate health inequalities through both direct and indirect pathways. From a policy perspective, these findings suggest that public interventions should be tailored to the specific characteristics of different territorial contexts, while maintaining consistent national standards and oversight. For example, in northern areas where economic disparities play a decisive role, policies aimed at reducing economic inequality, such as tax incentives and enhanced social support networks, could strengthen the protective effect of education on health. In contrast, in central and southern regions, alongside economic initiatives, greater emphasis should be placed on promoting physical activity and fostering community participation, consistent with evidence on the importance of social connections (19, 36). Crucially, these interventions must be implemented within a unified national framework that safeguards equal rights and prevents fragmentation of health services. Ultimately, our evidence advocates for a multidimensional and context-sensitive policy approach that balances regional specificity with national cohesion, aiming to reduce health inequalities and improve quality of life amid demographic aging.

## Data Availability

Publicly available datasets were analyzed in this study. This data can be found here: https://www.istat.it/en/microdata/multipurpose-survey-on-households-aspects-of-daily-life/.
