# Supplementary material for: Exploring the determinants of health in the aging population: the key role of education and socioeconomic context
Source: Front Public Health. 2026 Feb 26;14:1659900. doi: 10.3389/fpubh.2026.1659900 (PMC13006030; doi:10.3389/fpubh.2026.1659900)
Supplement: Supplementary file 1 [file Supplementary_file_1.docx]

### **Appendix**

Table A1. Descriptive statistics (N=94,916).

| *Health* | *%* |  | *Gender* | *%* |  | *Smoke* | *%* |
| --- | --- | --- | --- | --- | --- | --- | --- |
| Bad | 61.98 |  | Male | 43.40 |  | Yes | 43.27 |
| Good | 38.02 |  | Female | 56.60 |  | No | 56.73 |
|  |  |  |  |  |  |  |  |
| *Education* | *%* |  | *Age* | *%* |  | *Physical activity* | *%* |
| No title | 8.4 |  | 55-59 | 17.00 |  | Yes | 30.87 |
| Primary | 36.59 |  | 60-64 | 16.09 |  | No | 69.13 |
| Lower secondary | 27.12 |  | 65-74 | 31.32 |  |  |  |
| Upper secondary | 21.04 |  | +75 | 35.59 |  |  |  |
| Tertiary | 6.85 |  |  |  |  |  |  |
|  |  |  |  |  |  |  |  |
| *Geographical area* | *%* |  | *Labour market conditions* | *%* |  | *Marital status* | *%* |
| North-West | 21.40 |  | Employed | 17.12 |  | Single | 7.43 |
| North-East | 18.43 |  | Unemployed | 3.26 |  | Married | 62.33 |
| Centre | 18.81 |  | Not in labour force | 27.84 |  | Divorced | 6.76 |
| South | 30.45 |  | Retired | 51.79 |  | Widowed | 23.49 |
| Islands | 10.91 |  |  |  |  |  |  |
|  |  |  |  |  |  |  |  |
| *Alcohol consumption* |  |  |  |  |  |  |  |
| Mean | 0.638 |  |  |  |  |  |  |
| Std. dev. | 0.004 |  |  |  |  |  |  |

Table A2. Logistic regression models for probability of being healthy. Marginal effects and respective standard errors.

|  | Model 1 | | Model 2 | | Model 3 | |
| --- | --- | --- | --- | --- | --- | --- |
|  | Coeff. | S.E. | Coeff. | S.E. | Coeff. | S.E. |
| *Education* |  |  |  |  |  |  |
| No title | 0.000 | (.) | 0.000 | (.) | 0.000 | (.) |
| Primary | 0.075^***^ | (0.006) | 0.069^***^ | (0.006) | 0.070^***^ | (0.006) |
| Lower secondary | 0.121^***^ | (0.007) | 0.109^***^ | (0.007) | 0.112^***^ | (0.007) |
| Upper secondary | 0.168^***^ | (0.007) | 0.146^***^ | (0.007) | 0.154^***^ | (0.007) |
| Tertiary | 0.211^***^ | (0.009) | 0.176^***^ | (0.009) | 0.191^***^ | (0.009) |
|  |  |  |  |  |  |  |
| *Gender* |  |  |  |  |  |  |
| Male | 0.000 | (.) | 0.000 | (.) | 0.000 | (.) |
| Female | -0.035^***^ | (0.004) | -0.038^***^ | (0.004) | -0.022^***^ | (0.004) |
|  |  |  |  |  |  |  |
| *Age* |  |  |  |  |  |  |
| 55-59 | 0.000 | (.) | 0.000 | (.) | 0.000 | (.) |
| 60-64 | -0.007^*^ | (0.004) | -0.009^**^ | (0.004) | -0.010^**^ | (0.004) |
| 65-74 | -0.029^***^ | (0.004) | -0.032^***^ | (0.004) | -0.031^***^ | (0.004) |
| 75+ | -0.112^***^ | (0.005) | -0.119^***^ | (0.005) | -0.103^***^ | (0.005) |
|  |  |  |  |  |  |  |
| Marital status |  |  |  |  |  |  |
| Single | 0.000 | (.) | 0.000 | (.) | 0.000 | (.) |
| Married | 0.033^***^ | (0.006) | 0.029^***^ | (0.006) | 0.029^***^ | (0.006) |
| Divorced | 0.005 | (0.008) | 0.016** | (0.008) | 0.006 | (0.008) |
| Widowed | -0.003 | (0.007) | -0.002 | (0.007) | -0.001 | (0.007) |
|  |  |  |  |  |  |  |
| *Labour market condition* | | | | | | |
| Employed | 0.000 | (.) | 0.000 | (.) | 0.000 | (.) |
| Unemployed | -0.079^***^ | (0.009) | -0.050^***^ | (0.009) | -0.080^***^ | (0.009) |
| Not in labour force | -0.090^***^ | (0.006) | -0.082^***^ | (0.006) | -0.095^***^ | (0.006) |
| Retired | -0.072^***^ | (0.006) | -0.072^***^ | (0.006) | -0.081^***^ | (0.006) |
|  |  |  |  |  |  |  |
| *Wave* |  |  |  |  |  |  |
| 2013 | 0.000 | (.) | 0.000 | (.) | 0.000 | (.) |
| 2014 | -0.001 | (0.006) | -0.004 | (0.006) | -0.001 | (0.006) |
| 2015 | 0.011^**^ | (0.006) | 0.006 | (0.006) | 0.012^**^ | (0.006) |
| 2016 | 0.001 | (0.006) | -0.007 | (0.006) | 0.003 | (0.006) |
| 2017 | 0.024^***^ | (0.005) | 0.014^***^ | (0.005) | 0.026^***^ | (0.005) |
| 2018 | 0.001 | (0.006) | -0.008 | (0.006) | 0.004 | (0.006) |
| 2019 | 0.014^**^ | (0.006) | 0.002 | (0.006) | 0.016^***^ | (0.006) |
|  |  |  |  |  |  |  |
| *Geographical area of residence* | | | | | | |
| North-West | 0.000 | (.) | 0.000 | (.) | 0.000 | (.) |
| North-East | 0.038^***^ | (0.005) | 0.034^***^ | (0.005) | 0.031^***^ | (0.005) |
| Centre | -0.020^***^ | (0.005) | -0.017^***^ | (0.005) | -0.013^***^ | (0.005) |
| South | -0.076^***^ | (0.004) | -0.070^***^ | (0.004) | -0.059^***^ | (0.004) |
| Islands | -0.075^***^ | (0.006) | -0.066^***^ | (0.006) | -0.054^***^ | (0.006) |
|  |  |  |  |  |  |  |
| *Economic strain* | | | | | | |
| No |  |  | 0.000 | (.) |  |  |
| Yes |  |  | -0.095^***^ | (0.003) |  |  |
|  |  |  |  |  |  |  |
| *Smoke* |  |  |  |  |  |  |
| No |  |  |  |  | 0.000 | (.) |
| Yes |  |  |  |  | -0.029^***^ | (0.003) |
|  |  |  |  |  |  |  |
| *Physical activity* |  |  |  |  | 0.000 | (.) |
| *No* |  |  |  |  | 0.093^***^ | (0.003) |
| Yes |  |  |  |  |  |  |
|  |  |  |  |  |  |  |
| *Alcohol* |  |  |  |  | 0.038^***^ | (0.003) |
| *Alcohol ^2^* |  |  |  |  | -0.005^***^ | (0.001) |
| Observations | 94,916 |  | 94,916 |  | 94,916 |  |
| Pseudo *R*^2^ | 0.078 |  | 0.085 |  | 0.087 |  |

* p < 0.10, ** p < 0.05, *** p < 0.01
